# Antiviral and Immunomodulatory Effects of Phytochemicals from Honey against COVID-19: Potential Mechanisms of Action and Future Directions

**DOI:** 10.3390/molecules25215017

**Published:** 2020-10-29

**Authors:** Mohammad A. I. Al-Hatamleh, Ma’mon M. Hatmal, Kamran Sattar, Suhana Ahmad, Mohd Zulkifli Mustafa, Marcelo De Carvalho Bittencourt, Rohimah Mohamud

**Affiliations:** 1 Department of Immunology, School of Medical Sciences, Universiti Sains Malaysia, Kubang Kerian 16150, Kelantan, Malaysia; alhatamleh@student.usm.my (M.A.I.A.-H.); suhanaahmad1207@gmail.com (S.A.); 2 Department of Medical Laboratory Sciences, Faculty of Applied Health Sciences, The Hashemite University, Zarqa 13133, Jordan; mamon@hu.edu.jo; 3 Department of Medical Education, College of Medicine, King Saud University, Riyadh 11472, Saudi Arabia; drkamransattar@gmail.com; 4 Department of Neurosciences, School of Medical Sciences, Universiti Sains Malaysia, Kubang Kerian 16150, Kelantan, Malaysia; zulkifli.mustafa@usm.my; 5 Hospital Universiti Sains Malaysia, Kubang Kerian 16150, Kelantan, Malaysia; 6 Université de Lorraine, CNRS, UMR 7365, IMoPA, F-54000 Nancy, France; marcelo.decarvalho@univ-lorraine.fr; 7 Université de Lorraine, CHRU-Nancy, Laboratoire d’Immunologie, F-54000 Nancy, France

**Keywords:** SARS-CoV-2, 2019-nCoV, antiviral agent, antiviral activity, antiviral immunity

## Abstract

The new coronavirus disease (COVID-19), caused by severe acute respiratory syndrome coronavirus-2 (SARS-CoV-2), has recently put the world under stress, resulting in a global pandemic. Currently, there are no approved treatments or vaccines, and this severe respiratory illness has cost many lives. Despite the established antimicrobial and immune-boosting potency described for honey, to date there is still a lack of evidence about its potential role amid COVID-19 outbreak. Based on the previously explored antiviral effects and phytochemical components of honey, we review here evidence for its role as a potentially effective natural product against COVID-19. Although some bioactive compounds in honey have shown potential antiviral effects (i.e., methylglyoxal, chrysin, caffeic acid, galangin and hesperidinin) or enhancing antiviral immune responses (i.e., levan and ascorbic acid), the mechanisms of action for these compounds are still ambiguous. To the best of our knowledge, this is the first work exclusively summarizing all these bioactive compounds with their probable mechanisms of action as antiviral agents, specifically against SARS-CoV-2.

## 1. Introduction

Coronavirus disease 2019 (COVID-19) was confirmed as a public health emergency by the World Health Organization (WHO) on 30 January 2020, as the coronavirus occurrence spread well beyond China, where it first emerged [1]. In early December 2019, many cases were diagnosed with pneumonia, with unidentified cause arisen in Wuhan, Hubei, China [1]. High-throughput sequencing from lower respiratory tract samples discovered 2019-nCoV, which was later called severe acute respiratory syndrome coronavirus-2 (SARS-CoV-2). According to a situation report released by WHO on 14 September 2020, SARS-CoV-2 has infected more than 28 million people globally and caused more than 900 thousand deaths [2]. This pandemic presents challenges and concerns for the majority of countries worldwide.

The common predecessor of all coronaviruses dates back as far as 55 million years or more, hence, suggesting elongated term coevolution with bat and avian species [3]. Existing historical evidence notifies that coronaviruses were initially discovered in the 1930s, when an acute respiratory contagion of house chickens was initiated by infectious bronchitis virus (IBV) [4]. Moreover, humanoid coronaviruses were first reported in the 1960s [5]. With the help of technology advancement, it was possible to learn the structure of coronavirus as enormous pleomorphic sphere-shaped elements with bulbous surface projections [6]. Furthermore, the typical width of the virus particles is around 120 nm (0.12 μm). The thickness of the envelope is ~80 nm (0.08 μm), and the spikes are ~20 nm (0.02 μm) long. Electron micrographs helped to identify the virus with an envelope comprising diverse pair of electron dense shells [7], and this envelope is a lipid bilayer where the sheath, envelope, and spike physical proteins are affixed [8]. Within this envelope, there is nucleocapsid comprised of manifold nucleocapsid (N) protein, bound to the positive-sense single-stranded RNA genome in an uninterrupted beads-on-a-string variety conformation [7].

**Figure 1:**
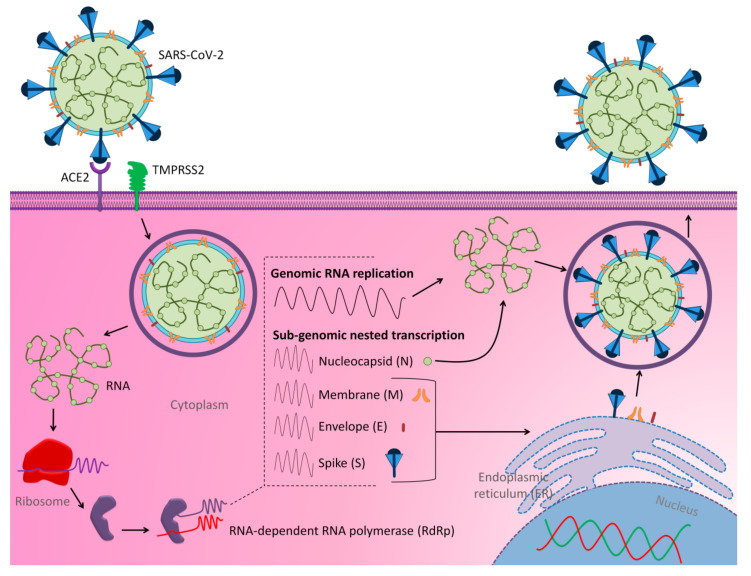
The schematic mechanism of replication of SARS-CoV-2 into host cell. Spike (S) protein on the surface of severe acute respiratory syndrome coronavirus-2 (SARS-CoV-2) recognizes the ACE2 receptor on the cellular membrane of host cell. After receptor binding, the virus enters host cell cytosol via cleavage of S protein by transmembrane protease/serine subfamily member 2 (TMPRSS2), followed by fusion of the viral and cellular membranes. The conformational changes at the S1 and S2 subunits facilitate the virus–cell fusion via the endosomal pathway. The viral genome is released into the cytoplasm and translated through the ribosomal frame, shifting to generate replicas polyproteins pp1a and pp1b. Negative-sense RNA intermediates are generated to serve as the templates for the synthesis of positive-sense genomic RNA (gRNA) and sub-genomic RNAs (sgRNAs). The gRNA is packaged by the structural proteins to assemble progeny virions. Shorter sgRNAs encode conserved structural and accessory proteins. Following gRNA and sgRNA synthesis, the viral proteins and genome RNA are inserted into virions and assembled in the ER-Golgi intermediate compartment (ERGIC) and then transported in the vesicle to the plasma membrane before releasing out via exocytosis pathway [12,13,14,15,16].

The SARS-CoV-2 spreads through droplets of saliva or discharges from the nose when infected persons cough or sneeze [9]. Angiotensin-converting enzyme 2 (ACE2) has been reported as a cellular receptor for SARS-CoV-2 [10]. ACE2 is expressed in a wide variety of human cells, including the type I and II alveolar epithelial cells in the lung [11]. During COVID-19 infection, the trimeric spike (S) glycoprotein on the SARS-CoV-2 surface mediates receptor recognition and membrane fusion, and it is cleaved into S1 and S2 subunits [10]. The S1 subunit comprises of receptor-binding domain (RBD), through the interaction with peptidase domain (PD) from the ACE2, whereas S2 subunit mediates virus–cell membrane fusion [10]. Upon the S1-ACE2 interaction, another S2 cleavage is identified and broken down by host proteases (i.e., priming) called transmembrane protease/serine subfamily member 2 (TMPRSS2), and this phase is considered crucial during viral infection [10]. Subsequently, the replication cycle of SARS-CoV-2 occurs in the host cell as summarized in Figure 1.

To date, we know that COVID-19 is a respiratory ailment, with the majority of affected people developing only mild to moderate symptoms and improving without the need of intensive management [17]. However, individuals with previous comorbidities (e.g., cardiovascular disease, diabetes, chronic respiratory disease, and cancer) and those above 60 years of age are at a greater risk of developing severe clinical form [18], known as acute respiratory distress syndrome (ARDS). The most common symptoms for COVID-19 are moderate to high-grade fever, tiredness, and dry cough. Additional symptoms may also include shortness of breath, body aches, sore throat, and very few individuals also experience diarrhea, nausea or a runny nose [19]. SARS-CoV-2 attacks the respiratory tract causing similar clinical symptoms to SARS-CoV and the Middle East respiratory syndrome coronavirus (MERS-CoV) [20].

**Figure 2:**
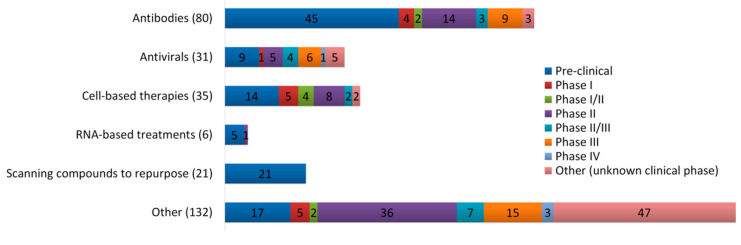
Classification of therapeutic substances being assessed in clinical trials, 305 trials in different stages, against COVID-19, according to ‘Milken institute COVID-19 treatment and vaccine tracker’ on 8 September 2020 [23].

So far, there is no approved vaccine or specific antiviral treatment against COVID-19, and WHO expects that effective vaccines will need at least 18 months of development before becoming available [21]. In order to fight against this disease, researchers have to repurpose and redevelop existing treatments to work against COVID-19 based on the molecular and biological understanding of the COVID-19 pathogeneses, which could be a promising option in terms of cost and time [22]. To date, several treatments have been used and some showed promising results but no definite curative effect has been confirmed. Thus, ascertaining effective treatments is imperative for patients’ benefit. Currently, there are 305 clinical trials to assess the therapeutic potentials of different types of drugs and other therapeutic compounds against COVID-19 and 194 of them reached the clinical stage (Figure 2).

Due to the current pandemic accelerating spread, and the length of time required for vaccine development and drug discovery, there is a pressing need for treatment and prevention strategies against COVID-19 based on existing natural products. Various natural products as potential treatment options for different types of infections have been extensively investigated and utilized. Among them, honey has long fascinated the attention of researchers as a complementary and alternative medicine [24]. Honey has religious and traditional histories from different ethnic communities worldwide in treating human diseases [25]. It has therapeutic properties as an antioxidant, anti-inflammatory, antibacterial, antimutagenic, antidiabetic, antifungal, antitumoral, antiviral and to expedite wound healings [24].

In an attempt to use honey as one of the treatments against COVID-19, antiviral properties of honey need to be exploited. Since ancient times, it is believed that honey is a valuable cure against pathogenic respiratory agents, including viruses that cause cough [26]. Several studies showed antiviral activity of honey against a wide range of viruses such as herpes simplex virus (HSV), human immunodeficiency virus (HIV), respiratory syncytial virus (RSV), varicella-zoster virus (VZV), adenovirus, and influenza viruses [27,28,29,30,31]. Furthermore, honey also possesses anti-inflammatory capacities and is recognized as a potent immune booster, which compliments it as an effective remedy to reduce the severity of viral diseases [32,33,34]. However, the therapeutic potential of honey against COVID-19 has still not been studied. Although many people believe that the antiviral effect of honey may work against SARS-CoV-2 and/or play an immunomodulatory role in COVID-19 patients, the potential mechanism of action still unclear. Therefore, it is worth clarifying these points scientifically, based on previous reports. In this review, we describe the potential effects of honey as a natural remedy to support our ongoing combat against COVID-19.

## 2. The Medicinal Properties of Honey

Although there are different types of honey from various producer bees, the chemical composition of 100 g of the commonly consumed honeys include approximately 64.9–73.1% carbohydrates, 35.6–41.8% fructose, 25.4–28.1% glucose, 16.9–18% water, 1.8–2.7% maltose, 0.23–1.21% sucrose, and 0.50–1% proteins, vitamins, amino acids, and minerals [35]. Honeys display variability in chemical composition associated with botanical and geographical origin, bee species, and climate [36]. The majority of the medicinal properties of honeys were associated with their antioxidant phenolic compounds that vary between honeys, typically based on the floral origin of the honey [35]. Phenolic compounds are plant secondary metabolites founded in honey with diverse chemical structures including phenolic acids and polyphenols (e.g., flavonoids). Despite the variability in the chemical composition of honeys, the most abundant flavonoids are apigenin, quercetin, luteolin, chrysin, kaempferol, galangin, genistein, pinocembrin, and pinobanksin, while the most abundant phenolic acids are gallic acid, chlorogenic acid, syringic acid, vanillic acid, *p*-coumaric acid, *p*-hydroxybenzoic acid, and caffeic acid [35].

Based on the floral source, there are monofloral (from a single floral source) and multifloral (from diverse floral sources) honeys. Most honeys are monofloral, produced commonly by bees from the genus *Apis*, and named according to their respective plant species (e.g., Manuka honey). Honeys produced by stingless bees (genus *Meliponinae*) are commonly multifloral [37]. However, which type of these two honeys can express superior therapeutic potentials (mainly based on its antioxidant activity) is still under investigation. A study of 10 different monofloral and multifloral honeys showed that the antioxidant activities, based on their phenolic content, of some monofloral honeys (i.e., heather > phacelia> honeydew > buckwheat) were higher compared to multifloral honeys, whereas other monofloral honeys (i.e., nectar–honeydew> lime > rape> goldenrods > acacia) showed lower antioxidant activities [38].

So far, the full process of absorption, metabolism, and excretion, which might be valid for all phenolic compounds, still requires clarification. Although the mechanisms behind the bioavailability of phenolic compounds have been addressed in few studies, only a few of these studies have specifically focused on those compounds derived from honey [39,40]. The utilization of phenolic compounds from honey in the clinical practice is often hampered by their very low bioavailability and absorption [41]. Understanding of the pharmacokinetics of phenolic compounds starts with phenol metabolism, which depends on hydrolysis reaction. This reaction can be performed by the lactase phlorizin hydrolase and the cytosolic β-glucosidase called β-endoglucosidase enzymes and are present in the small intestine [42]. These enzymes are responsible for catalyzing the β-hydrolysis of the sugar in the glycosylated phenolic compounds so they can be absorbed by the small intestine [43]. Some compounds contain sugars that prohibit the absorption but are deglycosylated by enzymes of microfloras presented in the colon. The final metabolites can either be absorbed or excreted through the feces or kidneys [43].

### 2.1. Honey as an Immune System Booster

**Figure 3:**
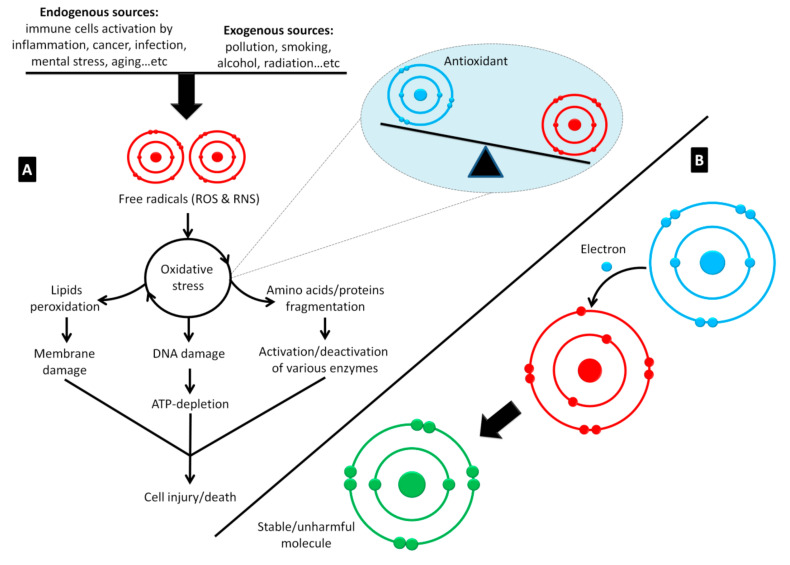
The principal of oxidative damage and the role of antioxidants in scavenging free radicals. (**A**) The free radicals generated from endogenous sources, at limited concentrations, are considered important for regulation of cell maturation, in addition to their role in immune defense. Excessive concentrations of these unstable molecules can result from illness conditions and exogenous sources, and thus lead to oxidative damage (imbalance between free radical and antioxidant concentrations). This status results in cell injury/death based on the extremely high reactivity of free radicals with vital cellular molecules including lipids, amino acids, proteins, and DNA. (**B**) Antioxidants are necessary to stop oxidative damage by neutralizing free radicals. They own this unique role due to their capability to give an electron to free radicals that have unpaired electrons to make them stable and unharmful. ROS, reactive oxygen species; RNS, reactive nitrogen species (adapted from Al-Hatamleh et al., 2020 [36]).

It is well-known that honey is an immune booster that improves the proliferation of T and B lymphocytes, stimulates phagocytosis, and regulates the production of vital pro-inflammatory cytokines from monocytes, such as tumor necrosis factor (TNF), interleukin 1 beta (IL-1β), and IL-6 [32,33]. On the other hand, honey also showed anti-inflammatory activity that inhibits the expression of these pro-inflammatory cytokines [34]. This dual immunomodulatory role of honey has been attributed to its antioxidant properties [34,44], which prevent and manage oxidative stress (Figure 3). The antioxidant activity of honey is positively correlated to its phenolic compounds content [45].

**Figure 4:**
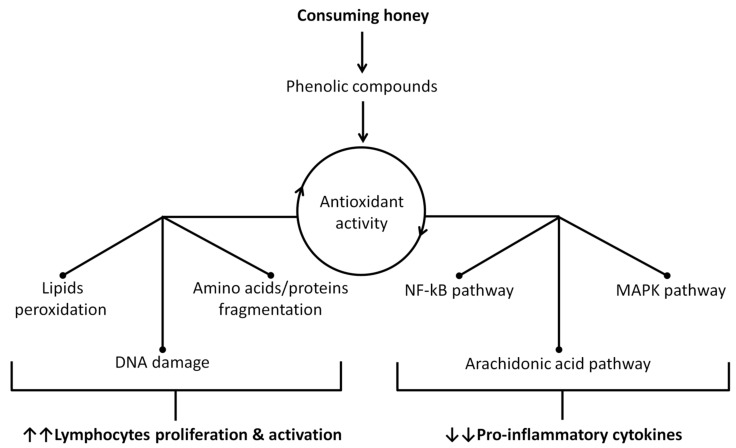
Potential mechanisms of action of honey as an immunomodulatory agent. These mechanisms relied on the antioxidant activity of honey. This activity inhibits oxidative stress and results in stopping harm to the vital cellular components (lipids, amino acids/proteins, and DNA), which promotes lymphocytes proliferation and activation. On the other hand, inhibition of the mitogen-activated protein kinases (MAPK) and the nuclear factor kappa-light-chain-enhancer of activated B cells (NF-kB) pathways results in complicated cellular mechanisms finished with suppression of pro-inflammatory genes, and thus blocks the expression of pro-inflammatory cytokines. In addition, the antioxidant activity also reduces the release of arachidonic acid, which results in the oxidation of membrane phospholipids, and thus reduces its metabolites (leukotrienes and prostaglandins) that are considered as important inflammatory mediators [34,49].

According to the current literature, the severity of COVID-19 infection correlates with lymphocytopenia, and patients who died from COVID-19 had lower lymphocyte counts compared to survivors [46,47]. These data suggest that lymphocyte-mediated antiviral activity is poorly effective against COVID-19. Despite lymphocytopenia, evidence for an exaggerated release of pro-inflammatory cytokines (i.e., IL-1 and IL-6) has been reported in the course of acute respiratory syndrome in COVID-19 infected patients, aggravating the clinical course of the disease [48]. Therefore, honey is anticipated to play a vital role in boosting the immune system as a supportive treatment for patients infected with COVID-19, and also for preventive measures for healthy individuals (Figure 4).

On the other hand, studies showed that antioxidants could modulate the signal transduction pathways crucial to cellular responses including inflammation, survival, cellular proliferation, and death, that are affected by oxidative stress [50,51]. For example, the nuclear factor-erythroid-2-related factor-2 (Nrf2) can be modulated by antioxidants, which results in the activation of some Nrf2 target gene candidates (e.g., Nrf2, SLC48A1, SLC7A11, p62, HO-1, and Bcl-2 genes) that control antioxidant defense and autophagy [52]. Furthermore, inhibition of phosphodiesterases (PDE), which can result from antioxidant activity, promotes the intracellular cyclic AMP (cAMP) second messenger system. Therefore, activation of cAMP response element-binding protein (CREB) targets genes and the AMP-activated protein kinase (AMPK) pathway, which is the key regulator of autophagy and is also involved in the regulation of Nrf2 pathway [52]. Altogether, the potential immune booster activities of antioxidants from honey are not only limited to inducing lymphocytes proliferation and activation and inhibiting the production of pro-inflammatory cytokines, it also can induce autophagy machinery. Thus, promoting these three immune responses could help to fight against COVID-19.

### 2.2. The Antiviral Activity of Honey

Although the antimicrobial activities of honey have been well studied against many bacteria and fungi [53,54], its antiviral activities still need an extensive exploration so that it can be used as prevention and treatment of viral infections.

In 1996, Zeina et al. suggested that honey has antiviral activity against the Rubella virus in infected monkey kidney cells (Vero cells) in vitro [55]. After four days of incubation, 1 mL of honey (at a range of concentrations from 1:1 to 1:1000) was enough to kill 1 mL of the virus in the culture in all concentrations (10 to 10^9^ virus/mL) without causing any cytotoxicity against the cells themselves [55]. At a concentration of 500 µg/mL, honey showed highest antiviral activity against HSV in vitro with a decrease in viral load at a concentration of 100 µg/mL [27]. Furthermore, honey is shown to be effective upon the topical use to treat recurrent skin lesions caused by HSV [56]. In addition, the antiviral activity of ‘Manuka’ (from mānuka tree flowers) and clover honeys against VZV has been reported in vitro [28]. Another study has shown that honeys including Manuka, Soba, Kanro, Acacia, and Renge have antiviral effects, and Manuka honey is the most potent antiviral candidate against influenza virus A/WSN/33 (H1N1) in the cultured Madin–Darby canine kidney (MDCK) cell line [29]. Moreover, extracts of honey, garlic, and ginger (HGG) mixture showed antiviral activity against influenza A virus isolates and is comparable to the standard antiviral drug Amantadine [57]. This in vitro study showed that HGG inhibited H1N2 replication in human peripheral blood mononuclear cells (PBMCs) and promoted cellular proliferation [57].

Previous reports on patients infected with HIV showed that consumption of honey helps to boost their immunity through the increase of lymphocytes proliferation, and generally improves their haematological and biochemical status (e.g., erythrocytes, haemoglobin, platelets, neutrophils, copper, and proteins levels) [58,59,60]. Meanwhile, another study also showed that consumption of honey in HIV positive subjects not only increases CD4 T lymphocytes counts but also decreases the viral load [61]. Other in vitro research on Manuka honey was carried out by Zareie, who examined its antiviral activity against RSV [31]. The inhibition and neutralization experiments showed a significant inhibitory effect on the progression of infection by honey through the inhibition of viral replication and the mRNA copy numbers of two viral genes [31]. It is reported that methylglyoxal, a compound in honey, serves as an antiviral agent for HIV [62,63]. Methylglyoxal affects the late stage of infection of HIV, where it blocks virion assembly and maturation [63].

It is known that the advanced glycation end-products (AGEs) of DNA and protein give rise to a major cell-permeant precursor “methylglyoxal”. Methylglyoxal reacts with free amino groups of lysine and arginine and with thiol groups of cysteine, forming AGEs [64]. Thus, higher levels of methylglyoxal were associated with dysfunctioning glyoxalase system, the most important pathway for the detoxification of methylglyoxal, which is related to various diseases including diabetes, cardiovascular disorders, cancer, and central nervous system problems [65]. Recently, studies have shown that the methylglyoxal content of honey is responsible for much of the honey’s antimicrobial properties. It was proven that methylglyoxal effectively inhibited the growth of gram-positive and gram-negative bacteria. These inhibitory effects were well-discovered, and it started when methylglyoxal levels reached 0.3 mM in media, causing alterations in the structure of bacterial fimbriae and flagella, which would limit bacteria adherence and motility [66]. However, there is no information precisely describing the mechanisms of activity for methylglyoxal against viruses. In the next section, the potential mechanisms of antiviral properties of honey are further discussed.

#### 2.2.1. MD-2/TLR4 Pathway

Toll-like receptor 4 (TLR4) is a transmembrane protein involved in the activation of host immune response upon pathogen infections via its interaction with myeloid differentiation protein 2 (MD-2) [67]. TLR4 is expressed by many cell types, although it is predominantly expressed by cells from the myeloid origin, mainly monocytes, macrophages and dendritic cells (DCs) [68]. Previously, it was known that only the lipopolysaccharide (LPS) from the outer membrane of gram-negative bacteria could activate the MD-2/TLR4 signaling axis based on the binding of LPS to the MD-2 hydrophobic pocket [69]. However, an increasing number of viruses show an activation of the inflammatory response mediated by the MD-2/TLR4 signaling axis as well [70].

**Figure 5:**
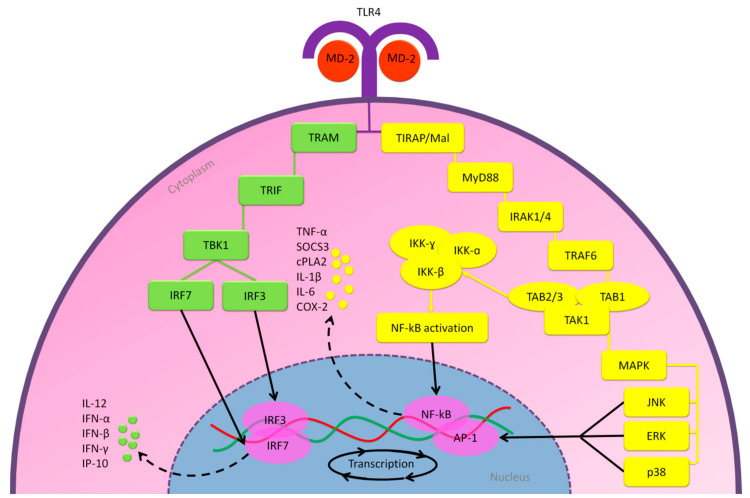
The signaling pathways of MD-2/TLR4 axis that lead to stimulating the immune responses. Through binding to MD-2 hydrophobic pockets, viral proteins activate the MD-2/TLR4 signaling axis. This interaction enables two cytoplasmic signaling domains; MyD88 via Toll–IL-1 receptor (TIR) domain-containing adaptor protein (TIRAP)/MyD88 adapter-like (Mal), and TIR domain-containing adaptor-inducing interferon-β (TRIF) via TRIF-related adaptor molecule (TRAM). The MyD88/TIRAP pathway uses the members of IL-1 receptor-associated kinases 1 and 6 (IRAK1/4), TNF receptor-associated factor 6 (TRAF6), and transforming growth factor beta-activated kinase 1 (TAK1) complex to activate two transcription factors; nuclear factor kappa B (NF-kB), through the I kappa B kinase (IKK) complex, and activator protein-1 (AP-1), through the mitogen-activated protein kinases (MAPK). Both NF-kB and AP-1 regulates gene expression in response to pathogen infections and controls cytokines expression. On the other side, the TRIF/TRAM pathway activates the transcription factor interferon regulatory factor 3 (IRF3) and IRF7, through TANK binding kinase 1 (TBK1), that is involved in the regulation of innate immune responses [80,81,82]. JNK, c-jun n-terminal kinase; ERK, extracellular signal-regulated kinase.

Published literatures revealed that the glycoprotein (GP) of the RNA Ebola virus (EBOV) could bind to the MD-2 hydrophobic pocket. Thus, it implicates the induction of the MD-2/TLR4 signaling axis and increases the expression of pro- and anti-inflammatory cytokines [71,72]. Another study showed that EBOV-GP was implicated in the induction of the severity of infection and T lymphocyte death [73]. Supporting results from other researchers demonstrated that the GPs of vesicular stomatitis virus (VSV), the fusion (F) protein of RSV, and the nonstructural protein 1 (nsp1) of dengue virus (DENV) are also involved in the activation of MD-2/TLR4 signaling axis [70,74,75]. Recently published work demonstrated that SARS-CoV-2 also causes excessive inflammatory responses [76], where it infects T lymphocytes through the S protein-mediated membrane fusion [77] and leads to lymphocytopenia, associated with the mortality rate of COVID-19 [78,79]. However, it is still unclear whether SARS-CoV-2 can replicate the infected T lymphocytes. Furthermore, SARS-CoV-2 has nsps 1–16 that results from cleavage of the polyproteins pp1a and pp1ab, as well as three structural glycoproteins, including S, envelope (E), and membrane (M) proteins [10]. This evidence might indicate that acute inflammatory response among patients infected by SARS-CoV-2 might at least partly result from activation of the MD-2/TLR4 signaling axis. However, so far, there are no studies reported on the direct interaction of SARS-CoV-2 and TLR4. The molecular mechanisms of the MD-2/TLR4 signaling pathway are presented in Figure 5.

Furthermore, it has been proposed that TLR4 activation could be beneficial for the viruses during viral infection, especially for those RNA viruses with a high mutation rate [70]. This advantage might be attributed by induction of host factors that encourage viral replication or suppress those that impede antiviral response [70]. On the other hand, TLR4 antagonists have shown suppressive inflammatory effects in several animal models of viral infections, including influenza viruses (have a close mechanism of coronaviruses); these effects have been remarked by the decreased production of cytokines and chemokines, in addition to relieved disease symptoms [75,83,84,85,86]. Furthermore, the study of TLR4 knockout mice indicated that TLR4 activation is also required for the innate immune defenses against virus invasion [70]. Activation of TLR4 was positively associated with activation of phosphatidylinositol-4,5-bisphosphate 3-kinase (PI3K) during some viral infections, including SARS-CoV [87,88].

Levan polysaccharide is a product from the fermentation process of *Bacillus subtilis* and it has been reported that levan can mediate the activation of TLR4 pathway and results in an increase of the inflammation process [89]. A study on *B. subtilis* isolated from honey showed that the biological activity of levan (β-2,6-fructan) produced by these bacteria have antiviral activity against the pathogenic respiratory RNA virus avian influenza (HPAI) A (H5N1) and the enteric DNA adenovirus type 40 [30]. Both H5N1 and SARS-CoV are RNA viruses that cause severe viral pneumonia leading to ARDS [90] and both viruses have the potential to cause global pandemics [91]. Thus, it is crucial to continuously explore potential therapeutics against these viruses, and levan might be a promising compound in honey. Therefore, it would be interesting to evaluate the potential of TLR4-mediated effects from levan in honey to balance the pro-inflammatory versus antiviral effect in patients infected by SARS-CoV2. Moreover, a study using the fish model suggested that levan can facilitate the aggregation of cells and viruses, and thus enhances the phagocytosis process [92], but this approach may still require further investigation.

#### 2.2.2. Nitric Oxide Pathway

Another interesting potential of honey as antiviral could be demonstrated through the nitric oxide (NO) pathway. It has been reported that honey elevates NO, an essential cellular neurotransmitter in several physiological processes [58,93]. It has also been suggested that NO has effective properties in some pathological conditions, including viral infections [94]. The emerging biological functions of the NO pathway that induce innate immunity have encouraged researchers to examine the potential antiviral effect of NO in the early 1990s [95].

A review published in 1998 disclosed that several in vivo and in vitro studies discovered the potential antiviral effect of NO on RNA and DNA viruses [95]. Lane et al. suggested that NO was able to block the replication of murine coronavirus (M-CoV), a group II coronavirus, in an infected OBL21 neuronal cell line [96]. This result was supported by another study on the Japanese encephalitis virus (JEV), which showed that NO profoundly inhibits viral RNA synthesis, viral protein accumulation, and virus release from infected cells [97].

**Figure 6:**
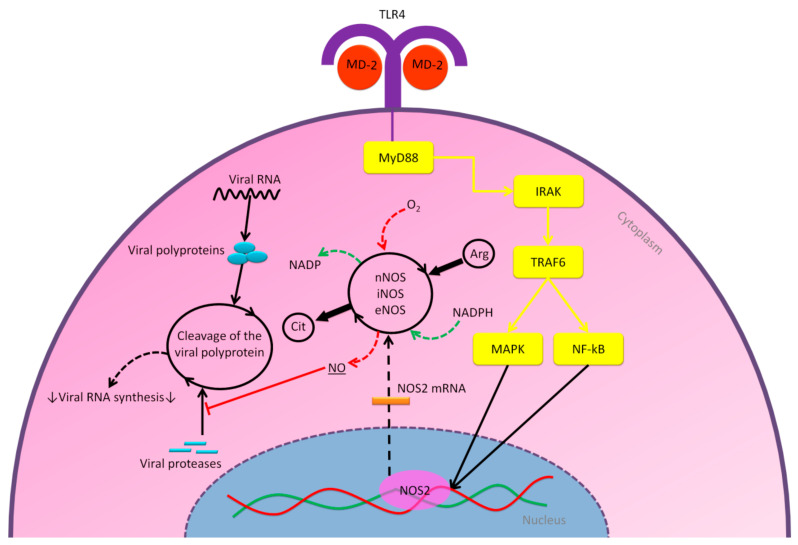
The hypothesized mechanisms of action for NO antiviral effect through the MD-2/TLR4 signaling axis, again implicated in the viral infection at the intracellular level. The MD-2/TLR4 signals activate the transcription of the nitric oxide synthase 2 (NOS2) gene through activation of MAPK and NF-kB. This activation results in expression of NOS2 mRNA to produce NOS enzymes that are responsible for producing NO by the conversion of l-arginine (Arg) into l-citrulline (Cit), with existing nicotinamide adenine dinucleotide phosphate (NADPH) and oxygen (O_2_). There are three isoforms from NOS enzymes that produce NO; neuronal NOS (nNOS), inducible NOS (iNOS), and endothelial NOS (eNOS); each of them is expressed in different cell types. In the cell infected by a virus, NO might inhibit viral protease enzymes by blocking their cleavage ability of viral polyproteins. This process would inhibit the synthesis of viral RNA, and thus inhibit viral replication.

In another study, researchers used NO donor *S*-nitroso-*N*-acetylpenicillamine (SNAP) for MDCK cells infected with influenza A and B viruses [98]. It was suggested that NO reduces infected cells productively and also impacts an early step in the viral RNA synthesis, consequently inhibiting virus production [98]. Although the mode of action of NO antiviral activity is still entirely unclear, especially against RNA viruses, studies indicate that NO likely targets viral enzymes [94,99,100]. In 2005, researchers showed that NO generated by inducible NO synthase enzyme, can inhibit the replication cycle of SARS-CoV in infected Vero E6 cells in vitro [101]. In this study, NO donor SNAP inhibited SARS-CoV replication in a dose-dependent manner from 100 μM to 400 μM, while the possibility that the antiviral effect resulted from general cytotoxicity was excluded and confirmed by MTT assay. Figure 6 illustrates the possible mechanism of action for NO antiviral activity.

An interesting case report showed that inhaled NO treatment (approved by the FDA) was used for the remote treatment of an outpatient infected with COVID-19 that had concomitant idiopathic pulmonary arterial hypertension (iPAH) [102]. Over 11 days, the inhaled NO dose was started from 20 ppm with oxygen for 12–14 h/day, and gradually decreased to 10, 5, and 0 ppm for 2–3 h/night. The patient had rapid and sustained improvement as evidenced by their symptomatic relief, and they recovered without hospital care [102]. However, the result of SARS-CoV-2 nucleic acid test after recovery was missed in this report, meaning it is unclear whether NO can express antiviral activity against SARS-CoV-2 or just prevent the progression of the disease. Therefore, several clinical trials are currently ongoing towards further understanding of the potentials of inhaled NO against COVID-19 [103].

## 3. Promising Insights for Honey Research amid COVID-19 Outbreak

So far, no published studies have observed the effects of honey on SARS-CoV-2. To the best of our knowledge, there are four registered clinical trials that are currently recruiting and even a completed study that assesses the efficacy of consuming honey and its active compounds in patients with COVID-19 (NCT04323345, NCT04345549, NCT04347382, NCT04468139). One of the trials aims to explore the efficacy of using 1 mg/kg of natural honey for 14 consecutive days compared with the standard health care protocols [104].

**Figure 7:**
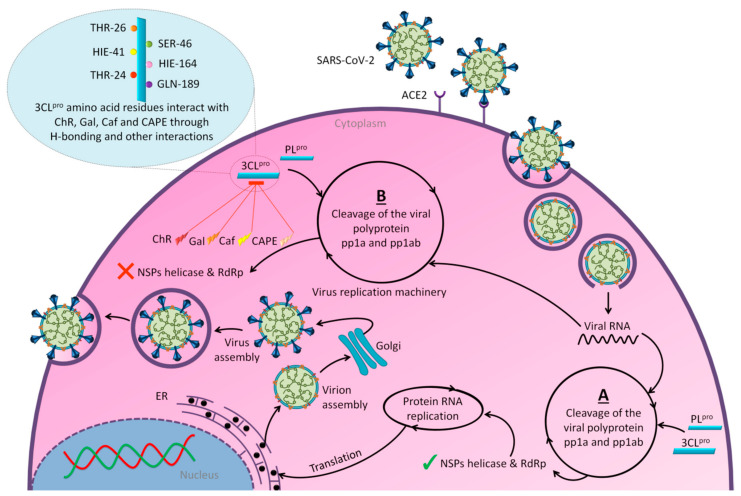
The potential antiviral mechanisms of action for honey contain caffeic acid, CAPE, galangin, and chrysin against SARS-CoV-2. Through an endosomal pathway, SARS-CoV-2 enters the host cell upon binding its S protein to the cellular receptor ACE2. (**A**) The viral RNA is unveiled in the cytoplasm and the pp1a and pp1ab polyproteins cleaved by the proteases (3CL^pro^ and PL^pro^) to form nonstructural proteins (nsps) as helicase (for viral RNA synthesis) and the RNA replicase–transcriptase complex (RTC), which includes RNA-dependent RNA polymerase (RdRp) (for virion assembly). RdRp is responsible for the replication of structural protein RNA. The nucleocapsids residing in the cytoplasm are assembled from genomic RNA, whereas the structural proteins S1, S2, E, and M are translated by ribosomes in the endoplasmic reticulum (ER), and then released for preparation of virion assembly. The structural proteins then fuse with virion assembly to virus assembling, which is then transported through the Golgi apparatus to be released via exocytosis. (**B**) When honey containing caffeic acid (Caf), CAPE, galangin (Gal), and chrysin (ChR) is being consumed, those compounds could enter the infected cells and inhibit 3CL^pro^. This inhibition is based on the chemical interactions of these compounds with 3CL^pro^ amino acid residues; (1) Caf with GLN-189, HIE-164 through hydrogen (H) bonding, and with HIE-41 through π–π stacking interaction. (2) CAPE with THR-24 and THR-26 through H-bonding, and with HIE-41 through π–π interaction. (3) Chr with SER-46, THR-24 and THR-26 through H-bonding, and with HIE41 through π–π interaction. (4) Gal with SER-46 and THR-24 through H-bonding, and with HIE-41 through π–π interaction. As a result, the process of pp1a and pp1ab polyprotein cleavage will fail to form nsps and RdRp, which means that protein replication cannot be completed and finally, SARS-CoV-2 replication will be stopped.

On April 13, 2020, an exciting preprint was posted on ChemRxiv by Heba Hashem, where the author had conducted an in silico analysis (molecular docking) to assess the potential effects of natural phenolic chemical compounds from honey against SARS-COV-2 [105]. Heba suggested that caffeic acid, caffeic acid phenethyl ester (CAPE), galangin, and chrysin have good potential to inhibit the viral 3-chymotrypsin-like cysteine protease (3CL^pro^) enzyme, and thus inhibit viral replication [105]. The overall concept of this hypothesis is extensively explained in Figure 7. However, these findings are still missing a supportive quantitative analysis on how much caffeic acid, CAPE, galangin, and chrysin are required to cause direct inhibitory actions on the SARS-CoV-2 proteases.

Antiviral activity of caffeic acid has previously been reported against HSV, poliovirus, and influenza A virus [106,107]. CAPE is shown to have antiviral activity against HIV and hepatitis C virus (HCV) [108], where galangin exhibits antiviral activity against HSV and coxsackie B virus type 1 (Cox B1) [109]. Chrysin is advocated as the most recommended among these antiviral compounds present in honey [29], as is its work against coxsackievirus B type 3 (CVB3), HSV (1 and 2) and enterovirus 71 (EV71), through inhibition of viral 3C-like main protease (3CL^pro^) activity [110,111]. Although none of these studies have assessed the antiviral effects of these compounds directly extracted from honey, an in vitro study assessed the role of caffeic acid, galangin, and chrysin in bee propolis against HSV-1 and confirmed their antiviral activities [112]. However, the exact mechanisms of these compounds for their antiviral activities, especially against RNA viruses, are still lacking.

It is worth mentioning that SARS-CoV-2 3CL^pro^ shares 99.02% sequence identity with 3CL^pro^ in MERS-CoV and SARS-CoV, and thus it is considered as a proven drug discovery target [113]. The potential inhibitory effect of chrysin has also been suggested previously by another computational analysis, and not only against the 3CL^pro^ but also against the second SARS-CoV-2 protease, papain-like protease (PL^pro^) [114]. It has been reported that SARS-CoV PL^pro^ is implicated in viral evasion of the innate immune responses by stripping ubiquitin and ISG15 from the infected cell proteins [115], thus suggesting that targeting SARS-CoV-2 PL^pro^ might potentially inhibit viral replication and immune evasion.

Both the flavonoid hesperidin and rosmarinic acid from plant extracts, which are also reported to be found in honey [116], have shown potential to inhibit SARS-CoV-2 3CL^pro^ in a study-based computational analysis [114]. Interestingly, the analysis showed that hesperidin was the only natural compound that can bind to the RBD of S protein, and thus it could neutralize ACE2 and spike-RBD binding [114]. Thus, honey contains hesperidin could have a potential effect in blocking the adhesion of the virus to the target cells. However, the study of those two flavonoids was based on their presence in plants as origin and no quantitative report is given as an example in honey. These findings should guide researchers to conduct further computational and experimental analysis towards a clear understanding of the potential of anti-SARS-CoV-2 agents in honey. This step would be a pivotal point to learn from the past and not repeat conventional studies reporting the effects of honey without understanding its mechanisms of action.

## 4. Future Directions

Hydrogen peroxide (H_2_O_2_) is also one of the constituents in honey reported to be responsible for its antimicrobial effects [117]. An in vitro study conducted in 1977 showed that H_2_O_2_ has strongly inactivated the human coronavirus 229E (HCoV-229E) and influenza viruses (A and B) [118]. Another study showed that H_2_O_2_ has inhibitory effects on the infectivity of bird viruses: H5N1, IBV, and Newcastle disease virus (NDV) [119]. A more recent study indicates the promising virucidal activity of H_2_O_2_ against the feline calicivirus (FCV), which infects domestic cats [120]. However, the antiviral activity of H_2_O_2_ in honey has not yet been assessed.

Ascorbic acid (vitamin C) is a common antioxidant that showed antiviral immune responses, especially against the influenza virus [121]. Although the familiar sources of ascorbic acid are fruit and vegetables, it is also one of the essential vitamins in some honeys [122]. Currently, there are four registered clinical trials using ascorbic acid as a treatment for patients infected with COVID-19 (NCT04354428, NCT03680274, NCT04344184, and NCT04357782), in addition to another clinical trial that uses it synergistically with hydroxychloroquine for prevention purposes in adults exposed to SARS-CoV-2 (NCT04328961) [123]. Additionally, *p*-coumaric acid, benzoic acid, and pinocembrin are also essential phenolic compounds present in commonly consumed honeys [35], and study of the therapeutic effect of bee propolis against HSV-1 has confirmed their antiviral activities [112]. Furthermore, the flavonoids isoquercetin, rutin, and quercetin are also constituents identified in honey [124]. A study on the influenza A virus (H5N1) has shown that each isoquercetin, rutin, and quercetin, isolated from the *Capparis sinaica* Veil (plant), has antiviral activity by reduction of H5N1 load, respectively [125]. The antiviral activities of all these compounds in honey are still undiscovered.

The described antiviral activity of honey could also be due to the fatty acid 10-Hydroxy-2-decenoic acid (10-HAD); it was proposed that 10-HAD induces the adhesion of leukocytes to viruses, resulting in their eradication [25]. It has been shown that 10-HAD promotes the maturation of dendritic cells (DCs) derived from human monocytes and the capability of T helper cell type-1 (Th1) polarization, which refers to a reinforcement in antiviral immunity [126]. Although the 10-HAD has only been reported in royal jelly (RJ) and not yet in other bee products (including honey) [127], another structure of fatty acids has been reported in both RJ and honey, and it is 3-hydroxy-sebacic acid (SEA) [128]. However, no studies to date have explored SEA effects on viruses or immunity. Table 1 shows all the potential antiviral compounds in honey and it could be a guide for future studies.

A study of virtual screening of some natural flavonoids from plant extracts against S protein has shown the high binding affinity of these compounds that is still undiscovered from honey, such as neohesperidin, licoflavonol, piceatannol, cosmosiin, excoecariatoxin, mangostin, phyllaemblicin, and kouitchenside D [114]. Since the diversity on phenolic compounds in honey depends on the vegetation at the site of the beehive [129], further research is required to assess the content of these effective compounds in honey as a potential anti-SARS-CoV-2 agent.

Synergistically, honey combinations with other rich natural products (such as cinnamon, garlic and ginger) have shown stronger antimicrobial and immune booster activities [25,57,130]. This approach could help to enhance the role of honey as a complementary and alternative medicine against COVID-19. Furthermore, in order to study the pharmacokinetic profiles of honey, an interesting study has shown that it can enhance the intestinal absorption of carboxyfluorescein as a drug model [131]. These findings refer to the potential of honey as a natural enhancer to improve the bioavailability of poorly absorbable drugs [131]. Thus, future studies should assess the probable effect of honey in enhancing the intestinal absorption of drugs being tested for repurposing to treat COVID-19. On the other hand, honey can be mediated by green synthesis (i.e., using natural products) of nanoparticles (NPs) [132] that show a promising role in controlling immune homeostasis during several inflammatory conditions [133,134,135]. NPs can also be used as a tool in rapid point-of-care diagnostics, surveillance, and monitoring, as well as in therapeutics delivery and vaccines development against COVID-19 [136].

Since not all honeys contain similar constituents, future studies should select honey with rich content of these compounds or any other potential anti-SARS-CoV-2 agents. Of note, studies have shown that honeys of stingless bees (found in tropical countries) are rich with the majority of those compounds that could have antiviral activities (Table 1), with high antimicrobial and medicinal properties [137,138]. To date, studies on the antiviral effects of honey have used only European/Western (*Apis*) honey. Although stingless bee honey is less used for consumption and scientific research, recent studies show that it could have higher nutritional and medicinal properties compared to the commonly used *Apis* honey [137,138]. Therefore, new research studies on the potential antiviral effects of stingless bee honey are necessary, not only against SARS-CoV-2, but also to explore its potential antiviral effects in general.

Nevertheless, the potential of honey against COVID-19 must be imperatively discussed. It is well established that COVID-19 clinically progresses through different stages [139]. In early stages of COVID-19 infection (stage I), a controlled viral response is induced and manifests mild, non-specific symptoms such as fever and dry cough. As the infection progresses, localized inflammation in the lung is norm (stage II) and a minority of patients would transition into a severe stage (stage III), which is manifested as systemic hyperinflammation. The potential of honey with its anti-inflammatory properties may benefit the later stage of COVID-19 infection. However, it needs to be noted that honey is also capable of inducing pro-inflammatory cytokines such as IL1β, TNF, and IL-6, associated with the systemic disease of COVID-19 infection [140], render its antiviral potential to be cautiously approached. Furthermore, its efficacy compared to conventional drugs such as glucocorticoids and remdesivir in the management of COVID-19 is very much lacking and similarly doubtful.

## 5. Conclusions

Overall, there are direct and indirect groups of evidence described in the literature referring to the opportunity of honey as a complementary therapy or preventive natural product amid COVID-19 outbreak. Consuming honey might help in reducing the severity of COVID-19 infection either directly based on its potential antiviral effects against SARS-CoV-2, or indirectly through boosting immune responses. The direct and indirect medicinal properties of honey against COVID-19 are mainly associated with its content of antioxidant phenolic compounds. However, despite honey benefits, it does not compensate seeking medical consultation and using medications. Further preclinical and clinical investigations are urgently needed to deeply explore the mechanisms of action for honey against COVID-19. A deeper and critical analysis of the pharmacokinetics of phenolic compounds derived from honey should also be performed.

## Figures and Tables

**Table 1:** Summary of the bioactive chemical compounds in honey that could have antiviral activities.

Compound	Discretion	Mechanisms of Antiviral Activities	Stage of Research	Reference
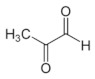 Methylglyoxal	Dicarbonyl resulted from the conversion of DHA during the ripening of honey	Blocks formation of virion assembly and maturation	In-vitro	[63]
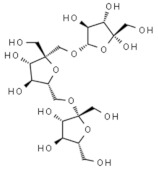 Levan	Polysaccharide produced by fermentation of *Bacillus subtilis*	Activation of antiviral immune responses	In-vitro	[30]
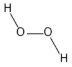 Hydrogen peroxide	Produced mainly during glucose oxidation	Viral inactivation	-	[118]
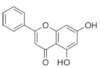 Chrysin	Flavonoid	Inhibition of viral protease enzymes	In-silico	[105]
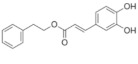 CAPE	Polyphenolic ester	Inhibition of viral protease enzymes	In-silico	[105]
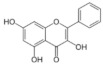 Galangin	Flavonoid	Inhibition of viral protease enzymes	In-silico	[105]
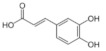 Caffeic acid	Flavonoid	Inhibition of viral protease enzymes	In-silico	[105]
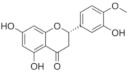 Hesperidin	Flavonoid	- Inhibition of viral protease enzymes - Binding to S-RBD and then blocking the interaction with ACE2	In-silico	[114]
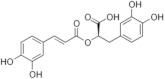 Rosmarinic acid	Polyphenolic hydroxycinnamic acid	Inhibition of viral protease enzymes	In-silico	[114]
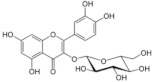 Isoquercetin	Flavonoid	Reduction of viral load	-	[125]
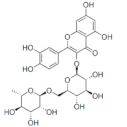 Rutin	Flavonoid	Reduction of viral load	-	[125]
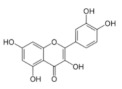 Quercetin	Flavonoid	Reduction of viral load	-	[125]
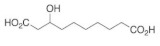 3-hydroxy-sebacic acid	Fatty acid	Unknown	-	[128]
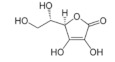 Ascorbic acid	Sugar acid	Activation of antiviral immune responses	-	[121]
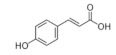 *p*-Coumaric acid	Phenolic acid	Unknown	-	[112]
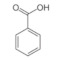 Benzoic acid	Aromatic carboxylic acid	Unknown	-	[112]
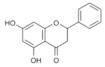 Pinocembrin	Flavonoid	Unknown	-	[112]

DHA, dihydroxyacetone; CAPE, caffeic acid phenylethyl ester; S-RBD, spike-receptor binding domain; ACE2, angiotensin-converting enzyme 2.
